# Proteomic analysis of brain metastatic lung adenocarcinoma reveals intertumoral heterogeneity and specific alterations associated with the timing of brain metastases

**DOI:** 10.1016/j.esmoop.2022.100741

**Published:** 2022-12-16

**Authors:** N. Woldmar, A. Schwendenwein, M. Kuras, B. Szeitz, K. Boettiger, A. Tisza, V. László, L. Reiniger, A.G. Bagó, Z. Szállási, J. Moldvay, A.M. Szász, J. Malm, P. Horvatovich, L. Pizzatti, G.B. Domont, F. Rényi-Vámos, K. Hoetzenecker, M.A. Hoda, G. Marko-Varga, K. Schelch, Z. Megyesfalvi, M. Rezeli, B. Döme

**Affiliations:** 1 Department of Biomedical Engineering, Lund University, Lund, Sweden; 2 Laboratory of Molecular Biology and Proteomics of Blood/LADETEC, Institute of Chemistry, Federal University of Rio de Janeiro, Rio de Janeiro, Brazil; 3 Department of Thoracic Surgery, Medical University Vienna, Vienna, Austria; 4 Section for Clinical Chemistry, Department of Translational Medicine, Lund University, Skåne University Hospital Malmö, Malmö, Sweden; 5 Division of Oncology, Department of Internal Medicine and Oncology, Semmelweis University, Budapest, Hungary; 6 National Korányi Institute of Pulmonology, Budapest, Hungary; 7 Department of Pathology and Experimental Cancer Research, Semmelweis University, Budapest, Hungary; 8 Department of Pathology, Forensic and Insurance Medicine, MTA-SE NAP, Brain Metastasis Research Group, Hungarian Academy of Sciences, Budapest, Hungary; 9 Department of Neurooncology, National Institute of Clinical Neurosciences, Budapest, Hungary; 10 Computational Health Informatics Program, Boston Children’s Hospital, Harvard Medical School, Boston, USA; 11 Danish Cancer Society Research Center, Copenhagen, Denmark; 12 Department of Bioinformatics, Semmelweis University, Budapest, Hungary; 13 Department of Analytical Biochemistry, Groningen Research Institute of Pharmacy, University of Groningen, Groningen, The Netherlands; 14 Department of Biochemistry, Institute of Chemistry, Federal University of Rio de Janeiro, Rio de Janeiro, Brazil; 15 Department of Thoracic Surgery, National Institute of Oncology-Semmelweis University, Budapest, Hungary

**Keywords:** lung adenocarcinoma, brain metastasis, clinical proteomics, intertumoral heterogeneity

## Abstract

**Background:**

Brain metastases are associated with considerable negative effects on patients’ outcome in lung adenocarcinoma (LADC). Here, we investigated the proteomic landscape of primary LADCs and their corresponding brain metastases.

**Materials and methods:**

Proteomic profiling was conducted on 20 surgically resected primary and brain metastatic LADC samples via label-free shotgun proteomics. After sample processing, peptides were analyzed using an Ultimate 3000 pump coupled to a QExactive HF-X mass spectrometer. Raw data were searched using PD 2.4. Further data analyses were carried out using Perseus, RStudio and GraphPad Prism. Proteomic data were correlated with clinical and histopathological parameters and the timing of brain metastases. Mass spectrometry-based proteomic data are available via ProteomeXchange with identifier PXD027259.

**Results:**

Out of the 6821 proteins identified and quantified, 1496 proteins were differentially expressed between primary LADCs and corresponding brain metastases. Pathways associated with the immune system, cell-cell/matrix interactions and migration were predominantly activated in the primary tumors, whereas pathways related to metabolism, translation or vesicle formation were overrepresented in the metastatic tumors. When comparing fast- versus slow-progressing patients, we found 454 and 298 differentially expressed proteins in the primary tumors and brain metastases, respectively. Metabolic reprogramming and ribosomal activity were prominently up-regulated in the fast-progressing patients (versus slow-progressing individuals), whereas expression of cell-cell interaction- and immune system-related pathways was reduced in these patients and in those with multiple brain metastases.

**Conclusions:**

This is the first comprehensive proteomic analysis of paired primary tumors and brain metastases of LADC patients. Our data suggest a malfunction of cellular attachment and an increase in ribosomal activity in LADC tissue, promoting brain metastasis. The current study provides insights into the biology of LADC brain metastases and, moreover, might contribute to the development of personalized follow-up strategies in LADC.

## Highlights

• Primary lung adenocarcinoma samples are enriched in immune system- and migration-related pathways.
• Brain metastases show upregulation of pathways associated with metabolism, translation and vesicle formation.
• Fast-progressing tumors are characterized by ribosome and metabolic pathways.
• Cell-cell interaction- and immune system-related pathways are diminished in fast-progressing tumors.
• MUC4, EPCAM, MRPL23 and MRPL47 might serve as potential predictors of fast-progressing brain metastases.

## Introduction

Non-small cell lung cancer (NSCLC) accounts for ∼85% of all lung cancer cases, with lung adenocarcinoma (LADC) being the most common NSCLC subtype (40% of total diagnoses).^2^ Treatment of NSCLC varies according to its histological features and disease stage.^3^, ^4^, ^5^ Adjustments in the management of advanced LADC have been facilitated by the interpretation of the genomic landscape, identification of novel biomarkers as well as the development of new therapeutic agents.^6^, ^7^, ^8^

Given the histological and cellular heterogeneity of LADC, and the considerable differences in therapeutic response, whole-genome and whole-exome sequencing studies have recently focused on identifying aberrant genes and structure variants to identify new personalized therapeutic approaches.^9^ Deletions or loss-of-function mutations in tumor suppressor genes such as *RB1*, *TP53* or *CDKN2A* together with recurrent alterations in *EGFR*, *ALK*, *PIK3CA*, *KRAS*, *BRAF* or *ERBB2* have been described as key molecular features in LADC.^10^, ^11^, ^12^ Importantly, targeted therapeutic agents focusing on some of these genetic alterations show increased efficacy when compared with conventional chemotherapy (ChT)^13^, ^14^, ^15^ Likewise, monoclonal antibodies such as nivolumab or pembrolizumab inhibit the interaction between programmed cell death protein 1 (PD-1) and programmed death-ligand 1 (PD-L1) and have demonstrated lasting therapeutic efficacy in patients with high PD-L1-expressing LADCs.^16^, ^17^, ^18^ Nevertheless, despite the rapid development of these novel therapeutic methods, considerable heterogeneity in clinical response still exists in NSCLC patients.

Distant NSCLC metastases are associated with significant morbidity, loss of functional independence and reduction in quality of life.^6^ Approximately 50% of all lung cancer patients have existing metastases at the time of initial diagnosis. As for their localization, the brain constitutes the most common distant metastatic site in LADC patients, followed by the bones.^19^, ^20^, ^21^ Notably, the incidence of brain metastasis (BM) can rise up to 50%-60% in patients harboring *EGFR* or *ALK* rearrangements over the course of their disease.^22^, ^23^, ^24^ Although cellular and molecular mechanisms underlying tumor progression have been extensively investigated in the past decade,^9^^,^^25^ early metastases represent a major barrier of therapeutic success. LADC is a dynamic disease and new mutations may also occur during disease progression, which explains its high degree of genetic heterogeneity. The molecular diversity between primary tumors and metastatic lesions, and moreover, the adaptation of clones to their environment, also significantly contribute to treatment failure.^26^ This highlights the need for appropriate early diagnostic and prognostic markers that help stratifying the patients for personalized therapeutic approaches.^27^

To date, only a few curative-intent treatment options exist for patients with BMs. While standard platinum-based ChT shows poor effectiveness due to its limited blood–brain barrier permeability,^28^ targeted agents (such as tyrosine kinase inhibitors) demonstrate therapeutic potency in BMs, but they are restricted to patients with a specific mutational landscape.^29^^,^^30^ Immune-checkpoint inhibitors might also represent an adequate treatment option for these patients,^31^ however, they display only moderate activity in PD-1 blockage of metastatic lesions in the central nervous system (CNS). Despite the homogeneity of driver mutations between the primary tumors and BMs, the proteome and immune microenvironment differ between tumor sites, thus impeding the overall therapeutic success.^32^

Exploring the proteomic landscape of primary tumors and corresponding BMs might provide insights into key driver proteins and signaling pathways of diagnostic and therapeutic importance. Nevertheless, due to the limited tissue availability, we have a rather limited knowledge of the extent to which BMs reflect the proteomic profile of the primary tumor in LADC patients. The aim of this study was to investigate the intertumoral heterogeneity in brain metastatic LADC patients with proteomic approaches, as well as to assess the impact of the existing proteomic pattern on the timing of BMs.

## Materials and methods

All reagents and details of the experimental procedures are described in Supplementary Materials and methods, available at https://doi.org/10.1016/j.esmoop.2022.100741. Sample preparation and data acquisition was conduced according to our previously described method.^1^ Data processing and statistical analysis workflow is presented in Supplementary Figure S1, available at https://doi.org/10.1016/j.esmoop.2022.100741.

### Tumor specimens

Formalin-fixed, paraffin embedded (FFPE) samples of the primary tumors were collected in the National Korányi Institute of Pulmonology, Budapest, Hungary whereas the corresponding BMs were received from the Department of Pathology and Experimental Cancer Research, Semmelweis University, Budapest, Hungary, both under informed written consent (ethical approval, 2521-0/2010-1018EKU). Patients who received neoadjuvant treatment before surgery or had a history of other malignant diseases in the last 5 years before lung cancer diagnosis were excluded from the study. Additionally, cases with extremely long intervals (>2000 days) between lung resection surgery and cerebral metastasectomy were excluded as well. According to the time between lung cancer diagnosis and BM surgery, patients were grouped either into fast-progressing (i.e. lung–brain interval ≤365 days) or slow-progressing (i.e. lung–brain interval >365 days) subgroups (Table 1). Of note, the cut-off value of 365 days for lung–brain intervals was selected based on the widely used incidence estimate thresholds for BMs in the clinics.^33^^,^^34^ Notably, some of the included patients developed multiple BMs during the course of their disease. Importantly, although these subsequent metastases were also removed surgically, we have only included the first BM of each patient in our analysis.

**Table 1:** Clinical and histopathological features within fast- and slow-progressing groups of primary LADC and corresponding BMs

Clinical parameters	Fast progression group (*n* = 11)	Slow progression group (*n* = 9)	
	Median (SD)	Median (SD)	*P* value^a^
Age at primary diagnosis (years)	61.5 (4.4)	57.5 (8.8)	0.8093
Time to brain metastasis (days)	247 (154.5)	1312 (484.7)	>0.0001
Overall survival (days)	611 (935)	2117 (674)	0.0012
Survival from brain surgery (days)	391 (1008)	638 (769)	0.4119

Gender (*n*)	Male	Female	Male	Female	*P* value^c^
	5	6	5	4	>0.9999

Smoking history (*n*)	Current	Former	Never	Current	Former	Never	*P* value^b^
	7	3	1	4	3	2	0.6184

	Yes/No	Yes/No	*P* value^c^
COPD (*n*)	5/6	1/8	0.1571
Multiple brain metastasis (*n*)	4/7	4/5	>0.9999

Histopathological characteristics		Fast progression group (*n* = 11)	Slow progression group (*n* = 9)	
		High/Low scores (n)	High/Low scores (n)	P value^c^
Mucin production	Prim	2/9	0/9	0.4789
	Met	3/8	0/9	0.2184
Stromal density	Prim	6/5	8/1	0.1571
	Met	6/5	7/2	0.3742
Necrosis	Prim	4/7	5/4	0.6534
	Met	8/3	6/3	>0.9999
Vascularization	Prim	6/5	4/5	>0.9999
	Met	11/0	9/0	>0.9999
Lymphatic score (density + distribution)	Prim	10/1	9/0	>0.9999
	Met	10/1	7/2	0.5658

		Mean area (SD)	Mean area (SD)	P value^a^
Tumor (%)	Prim	63.42 (33.96)	71.04 (29.06)	0.6550
	Met	83.23 (21.00)	68.61 (33.31)	0.2664
Adjacent tissue (%)	Prim	0	0	NA
	Met	4.65 (9.68)	9.95 (10.90)	0.2062

Median and standard deviation (SD) are presented for continuous variables, and number of patients (*n*) for categorical variables.

*P* values were calculated between fast- and slow-progressing subgroups.

Scores: 0-1 and 0-3 were considered low values, 2-3 and 4-6 were considered high values (see Supplementary Table S1, available at https://doi.org/10.1016/j.esmoop.2022.100741 for detailed information).

BM, brain metastasis; COPD, chronic obstructive pulmonary disease; LADC, lung adenocarcinoma; NA, not applicable.

a Mann–Whitney *U* test.

b χ^2^ test.

c Fisher’s exact test.

### Data availability

The mass spectrometry data have been deposited to the ProteomeXchange Consortium via the PRIDE^35^ partner repository with the data set identifier PXD027259.

## Results

### Clinicopathological characteristics of the patient cohort

In total, 20 patients with primary LADCs and corresponding BMs met the inclusion criteria (Table 1). Out of these, 11 patients featured early BMs (≤1 year), whereas 9 patients were classified into the slow-progressing (>1 year) subgroup. Importantly, in order to prevent any acute life-threatening complications, three patients from the fast-progressing subgroup were first treated for their BMs. The median overall survival was significantly different in the fast- versus slow-progressing subgroups (*P* = 0.0012) (Table 1 and Supplementary Figure S2, available at https://doi.org/10.1016/j.esmoop.2022.100741).

In primary LADC samples, histopathological evaluation revealed a mean tumor content of 63.42% and 71.04% in fast- versus slow-progressing patients, respectively (*P* = 0.6550). As for the BMs, the mean tumor content was 83.23% and 68.61% in patients with early versus late BMs, respectively (*P* = 0.2664) (Table 1). With regards to the surrounding tissue, primary lesions mainly displayed stromal parts and in two cases also necrosis outside of the tumor. In contrast, about half of the metastatic tumors showed adjacent necrosis and small areas with normal brain tissue rather than stroma. The different histological scores determined in each tissue specimen were classified into low- and high-value categories for statistical analysis. Necrotic, lymphocyte density and lymphocyte distribution scores as well as the ascertained lymphatic score did not reveal any differences when comparing fast- and slow-progressing patients (Table 1). Of note, significant differences in terms of tumoral vascularization were observed when comparing primary versus metastatic lesions (in fast-progressing *P* = 0.0351, in slow-progressing *P* = 0.0294). Specifically, BM samples showed higher levels of intratumoral vascularization in both progression groups. Detailed information about histopathological scores and patient characteristics are shown in Table 1 and Supplementary Table S1, available at https://doi.org/10.1016/j.esmoop.2022.100741.

### Proteomic patterns related to the histopathological features of LADC samples

**Figure 1:**
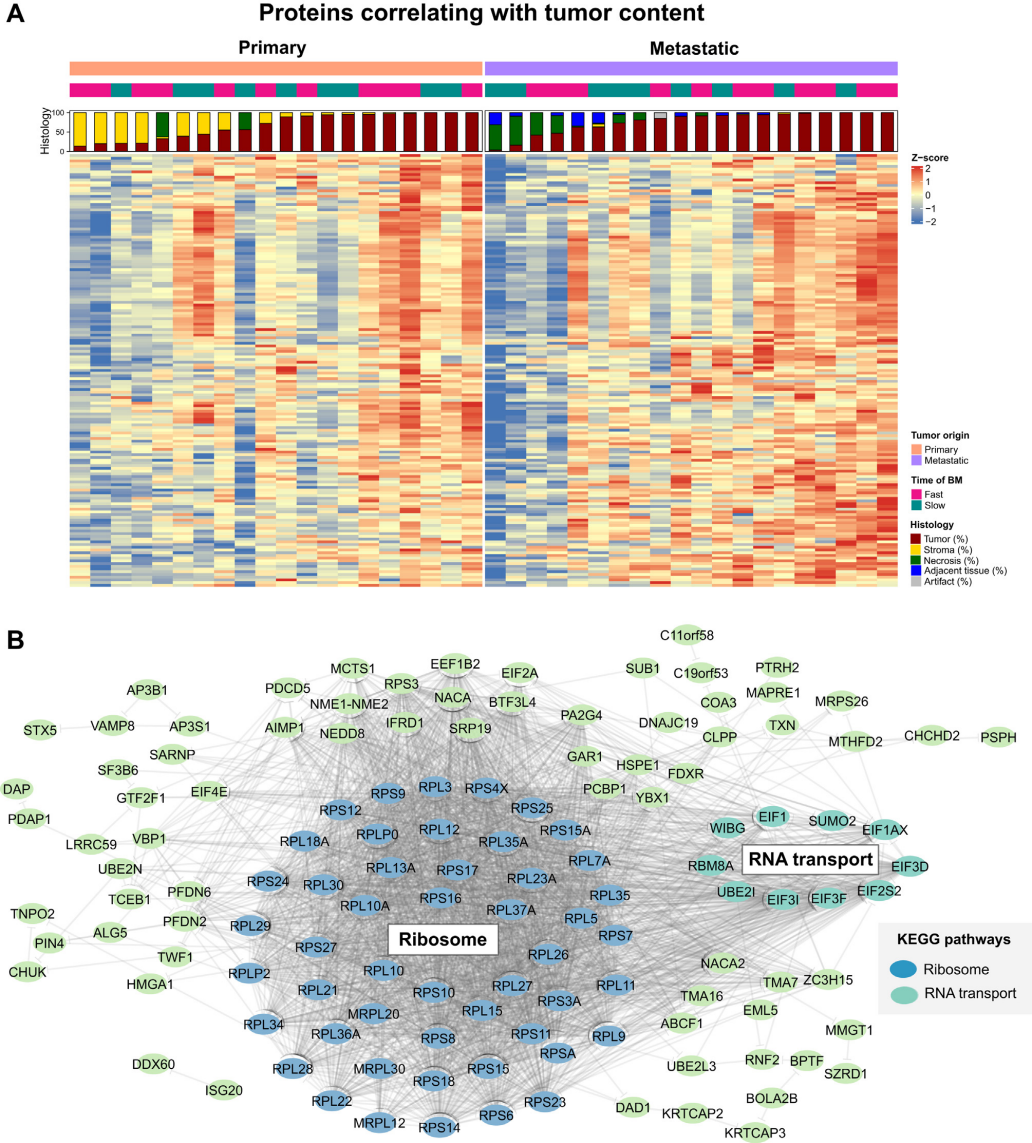
**Proteins positively correlating with tumor content in both primary and metastatic LADC tumors.** (A) Heatmap representation of the protein expression of 159 proteins that positively correlated with tumor content in primary and metastatic LADC tumors (*P* < 0.05; Spearman correlation). (B) Functional protein association network of the proteins positively correlating with tumor content in both primary and metastatic LADC samples. Proteins associated with the significantly enriched pathways, i.e. ribosome (green) and RNA transport (pink) are highlighted. BM, brain metastasis; LADC, lung adenocarcinoma.

Altogether, we identified and quantified 6821 proteins in the 20 primary tumors and corresponding BMs (Supplementary Table S1, available at https://doi.org/10.1016/j.esmoop.2022.100741). Their associated subcellular locations, biological processes, protein classes and molecular functions are shown in Supplementary Figure S3, available at https://doi.org/10.1016/j.esmoop.2022.100741. Notably, we found 466 and 996 proteins, which correlated positively with the tumor content in the primary and BM samples, respectively. Out of these, 159 were common both in the primary and metastatic lesions (Figure 1A). Looking into these overlapping proteins, we found 49 ribosomal proteins and 10 proteins involved in RNA transport (Figure 1B and Supplementary Table S2, available at https://doi.org/10.1016/j.esmoop.2022.100741). Importantly, when comparing this set of proteins with datasets from other studies (such as Gillette et al.^36^) we found that the vast majority of these proteins (∼70 %) were previously reported as up-regulated in LADC tumor samples in comparison to normal adjacent tissue (Supplementary Table S3, available at https://doi.org/10.1016/j.esmoop.2022.100741). These include ribosomal proteins L5, L10, L10a, L11, L15, L22 associated with tumor development and p53 activation,^37^, ^38^, ^39^ as well as other ribosomal proteins related to proto-oncogene/tumor suppressor regulation, cell malignant transformation, cell apoptosis regulation and cell growth or proliferation regulation (S3, S3a, S6, S27, L7a, L23a, L35a).^40^ Additionally, the Y-box binding protein 1 (YBX1), which has recently been described as a pro-metastatic gene,^41^ also correlated with the tumor content in our dataset.

In addition, we identified 97 proteins in the primary tumors and 76 proteins in the metastatic lesions which correlated positively with the presence and degree of intratumoral necrosis (i.e. necrotic score) (Supplementary Table S2, available at https://doi.org/10.1016/j.esmoop.2022.100741). Notably, seven of these proteins were common in both primary tumors and BMs, and two of the overlapping proteins are associated with the Rap1 signaling pathway, which is a key controller of cell-cell and cell-matrix interactions and is responsible for the regulation of mitogen-activated protein kinase (MAPK) activity.^42^

### Proteomic alterations between primary tumors and corresponding BMs

**Figure 2:**
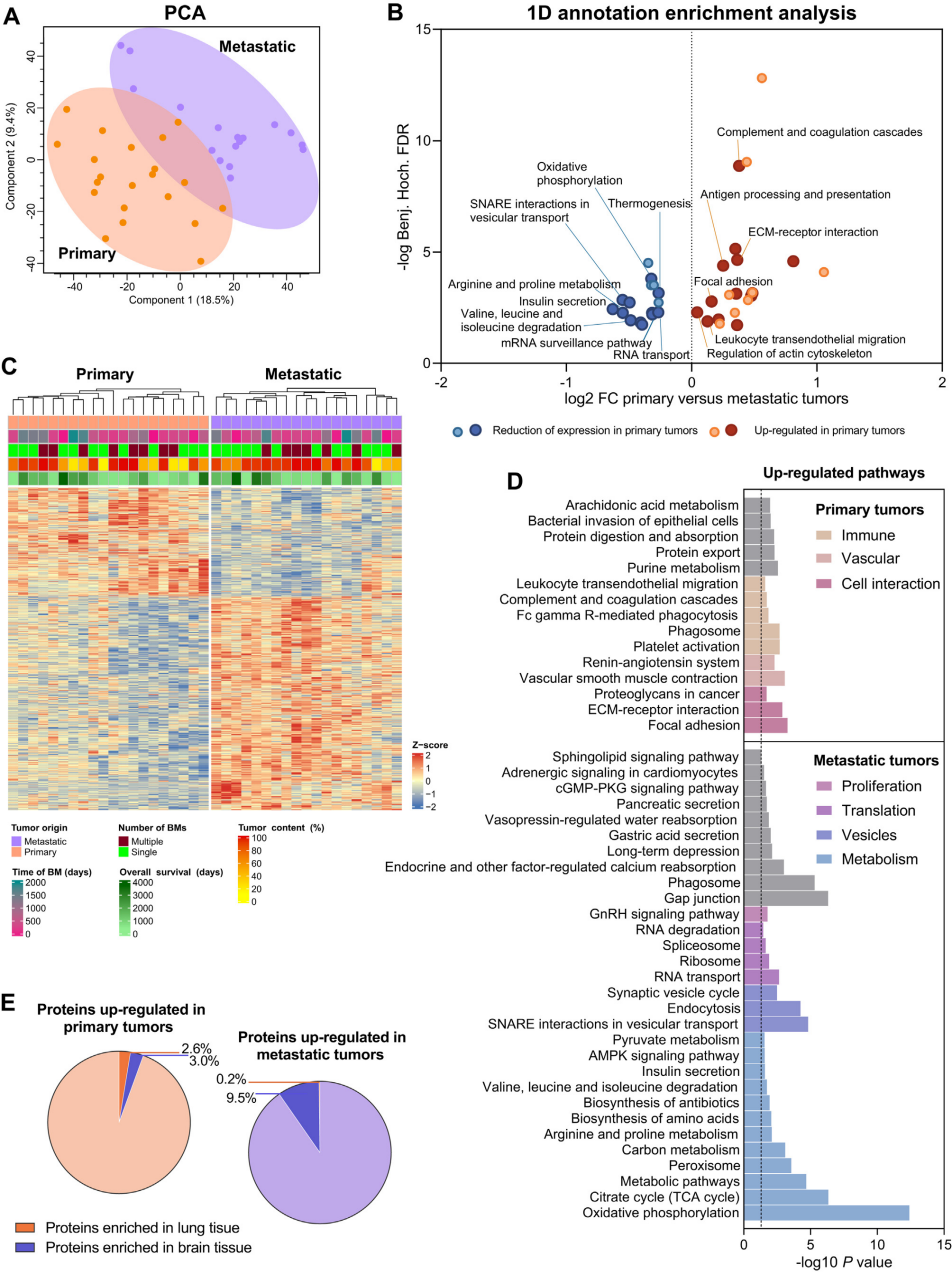
**Proteomic profiles associated with primary and metastatic LADC.** (A) PCA analysis of the whole cohort (excluding the outlier) showing two clusters according to their tissue of origin. Primary tumors are represented in light orange, whereas metastatic tumors are shown in light purple. (B) 1D annotation enrichment analysis illustrating the significant KEGG pathways [false discovery rate (FDR) < 0.02] up-regulated in primary (orange to red) or in metastatic (light to dark blue) LADC samples. (C) Heatmap representation of the significantly dysregulated proteins in primary versus metastatic LADC tumors (FDR < 0.05; *t*-test). (D) KEGG pathways associated with up-regulated proteins in primary (top) and metastatic tumors (bottom) (*P* < 0.05). Pathways with similar profiles were grouped in different colors. (E) Tissue-specific proteins identified among the up-regulated proteins in primary (dark orange) or metastatic (dark purple) LADC tumors using the Human Protein Atlas database. The lighter colors represent the non-tissue-specific proteins among the up-regulated proteins in primary (light orange) or metastatic (light purple) LADC tumors. AMPK, AMP-activated protein kinase; BM, brain metastasis; ECM, extracellular matrix; GnRH, gonadotropin-releasing hormone; LADC, lung adenocarcinoma; mRNA, messenger RNA; PCA, principal component analysis; PKG, protein kinase G; TCA, tricarboxylic acid.

Unsupervised clustering of the whole cohort mainly categorized the samples according to their tissue of origin (Supplementary Figure S4, available at https://doi.org/10.1016/j.esmoop.2022.100741, Figure 2A). Therefore, the differences in protein expression between primary and metastatic tumors were further analyzed. In addition, one BM sample (mLADC-19) displaying very low tumor content (3.6%) was classified as an outlier. Thus, it was excluded from further analyses (Supplementary Figure S1, available at https://doi.org/10.1016/j.esmoop.2022.100741, Figure 2A).

1D annotation enrichment analysis revealed that pathways associated with the immune system (e.g. complement and coagulation cascades, antigen processing and presentation and leukocyte transendothelial migration), cell-cell/matrix interactions and migration (e.g. extracellular matrix (ECM) receptor interaction, focal adhesion and regulation of actin cytoskeleton) were predominantly activated in the primary tumors (Figure 2B, Supplementary Table S4, available at https://doi.org/10.1016/j.esmoop.2022.100741). In comparison, pathways related to metabolism (e.g. oxidative phosphorylation, arginine and proline metabolism, insulin secretion and valine, leucine and isoleucine degradation), translation (e.g. messenger RNA (mRNA) surveillance and RNA transport) or vesicle formation (e.g. SNARE interactions in vesicular transport) were overrepresented in metastatic tumors.

Further investigation of protein expression differences between primary (*n* = 20) and metastatic (*n* = 19) LADC tissue samples resulted in 1496 differentially expressed proteins, of which 505 and 991 were up-regulated in the primary and metastatic samples, respectively (Figure 2C, Supplementary Table S1, available at https://doi.org/10.1016/j.esmoop.2022.100741). We identified only two proteins that were exclusively present in BMs (Supplementary Figure S5, available at https://doi.org/10.1016/j.esmoop.2022.100741). Enrichment analysis of the significantly up-regulated proteins in primary tumors revealed two vascular-related pathways (i.e. renin–angiotensin system and vascular smooth muscle contraction) in addition to the immune system-related, cell-cell/matrix interaction and migration pathways (Figure 2D). As for the metastatic tumors, pathways associated with metabolism, translation and vesicle formation were found to be enriched, reinforcing our findings gained by the 1D annotation enrichment analysis (Figure 2B and D). Besides these pathways, the gonadotropin-releasing hormone (GnRH) signaling pathway, which activates several downstream proliferation pathways, such as MAPK and epidermal growth factor (EGF), was also significantly up-regulated in BMs.

Differentially expressed proteins were thoroughly examined for tissue specificity using lung and brain tissue-specific protein databases from the Human Protein Atlas (HPA).^43^ The HPA datasets of lung- and brain-specific proteins, which encompass overexpressed proteins in the respective tissues, consist of 239 and 2587 proteins, respectively. Of the 505 significantly up-regulated proteins in the primary tumors, only 13 (2.6%) were lung-specific (Figure 2E, Supplementary Table S5, available at https://doi.org/10.1016/j.esmoop.2022.100741). Similarly, only 94 of the 991 significantly up-regulated proteins (9.5%) in the BM samples matched with the brain-specific protein dataset. Furthermore, we compared these up-regulated proteins with the human cancer metastasis database (HCMDB),^44^ and found an overlap of 19% and 10% on primary and metastatic levels, respectively (Supplementary Table S3, available at https://doi.org/10.1016/j.esmoop.2022.100741). These included a number of pro-metastatic gene products, such as COMP, TF, SFRP2, POSTN, CAV1, S100A4, LGALS1, COL6A1, CTSZ and HMGB1 overexpressed in primary tumors, and RAC1, SRC, YBX1, CSNK2A2, ENAH and GOLM1 overexpressed in metastatic tumor samples.^41^ We thus infer that the differentially expressed proteins between the primary LADC and the BM samples as well as their associated pathways are predominantly tumor tissue-related and not host tissue-specific.

### Proteomic features in LADC tissues associated with the timing of BMs

**Figure 3:**
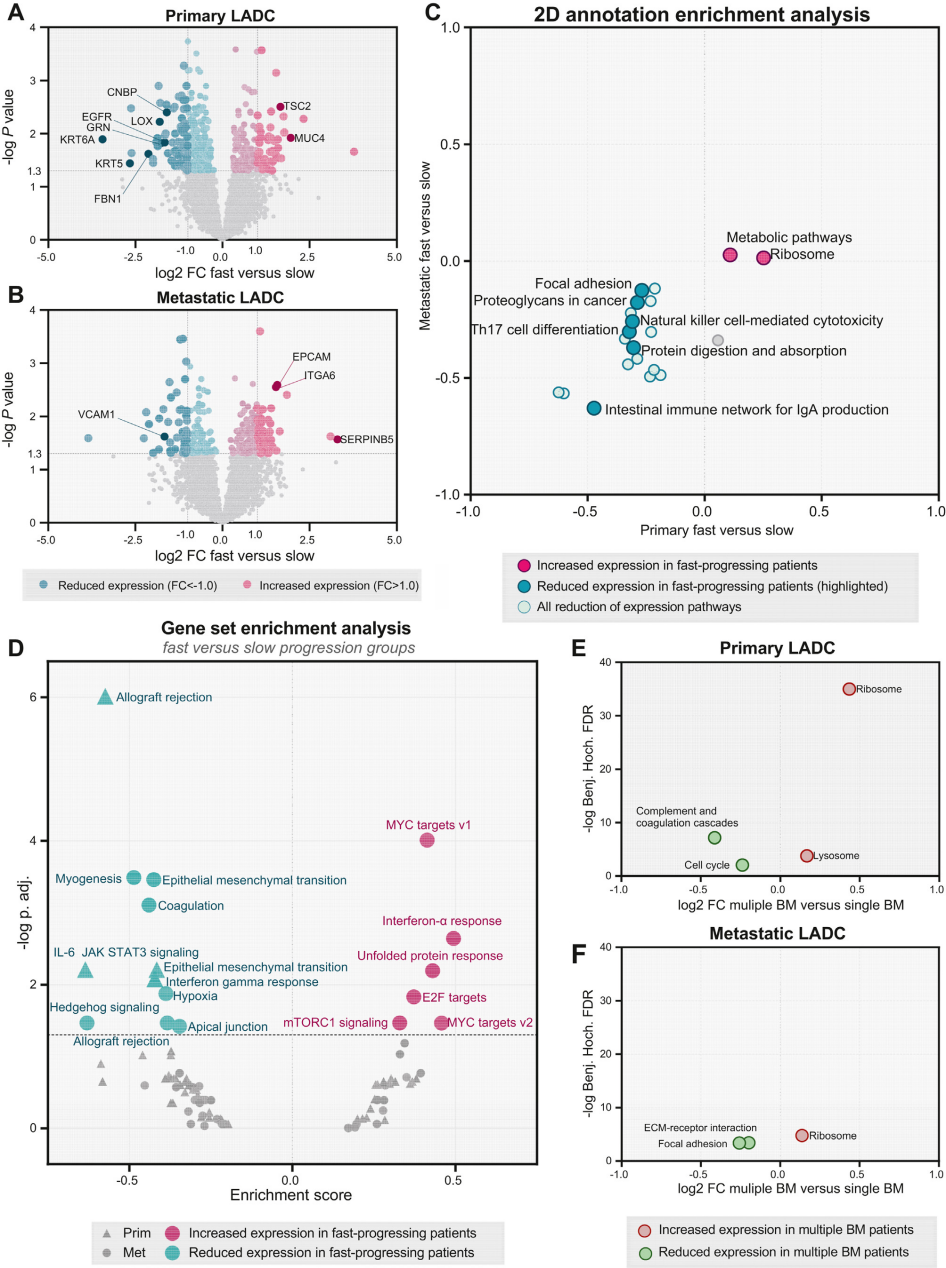
**Protein and pathway level comparison of patient samples according to the onset of brain metastasis.** Volcano plot showing proteins with significantly increased (pink) and reduced (turquoise) expression in (A) primary and (B) metastatic tumors of fast-progressing patients (*P* < 0.05; *t*-test). (C) 2D annotation enrichment analysis identified KEGG pathways commonly increased (pink) or reduced (turquoise) expression in primary and metastatic tumors of fast-progressing patients (FDR < 0.02). (D) Pre-ranked Gene Set Enrichment Analysis (GSEA) comparing fast- versus slow-progressing groups revealed significant dysregulated pathways in primary (triangles) and metastatic (dots) LADC tumors. 1D annotation enrichment analysis resulted in significant KEGG pathways with increased (red) or reduced expression (green) in (E) primary and (F) metastatic tumors of patients who developed multiple BMs (FDR < 0.02). BM, brain metastasis; FDR, false discovery rate; IgA, immunoglobulin A; IL-6, interleukin 6; LADC, lung adenocarcinoma

In order to investigate the potential causes and drivers behind the early development of BMs, we compared the proteomic landscape of fast- versus slow-progressing subgroups. This comparison was carried out separately on primary (fast *n* = 11 and slow *n* = 9) and metastatic (fast *n* = 11 and slow *n* = 8) tumor tissues and resulted in 454 and 298 differentially expressed proteins, respectively (Figure 3A and B, Supplementary Table S1, available at https://doi.org/10.1016/j.esmoop.2022.100741). The vast majority of differentially expressed proteins were identified in all the compared groups. In primary tumors, we found four and five on-off proteins in the fast- and slow-progressing groups, respectively, whereas in metastatic tumors we identified only two proteins that were exclusively expressed in the fast-progressing group (Supplementary Figure S5, available at https://doi.org/10.1016/j.esmoop.2022.100741). In primary tumors, the ribosome and metabolic pathways were among the top pathways associated with up-regulated proteins in the fast-progressing subgroup, whereas the proteins with reduced expression in this subgroup were primarily linked to pathways such as antigen processing and presentation, NOD-like receptor signaling, as well as focal adhesion, gap junction and Rap1 signaling. Up-regulated proteins in BMs of the fast-progressing subgroup were mainly involved in RNA transport, protein processing in endoplasmic reticulum, oxidative phosphorylation and metabolic pathways, whereas proteins with reduced expression were related to the lysosome, and vascular endothelial growth factor (VEGF) and MAPK signaling pathways (Supplementary Table S4, available at https://doi.org/10.1016/j.esmoop.2022.100741). In the samples of fast-progressing patients, we identified a number of proteins the expression of which increased more than two-fold compared with the slow-progressing subgroup, several of which were previously reported in the HCMDB (such as TSC2 and MUC4 in primary tumors, as well as EPCAM, ITGA6 and SERPINB5 in metastatic samples) (Figure 3A and B, Supplementary Table S3, available at https://doi.org/10.1016/j.esmoop.2022.100741). Nevertheless, many proteins with reduced expression in these patient samples have previously been described as related to metastatic spread.^44^

Subsequently, 2D annotation enrichment analysis, which is suitable for comparing two quantitative proteomic datasets, was used to identify KEGG pathways that show consistent behavior in fast- or slow-progressing groups regardless of tissue origin (Supplementary Table S4, available at https://doi.org/10.1016/j.esmoop.2022.100741). Accordingly, we found that the ribosome and metabolic pathways were up-regulated in the fast-progressing patients (versus slow-progressing patients) both in the primary and metastatic samples (Figure 3C). In contrast, expression of pathways such as focal adhesion, proteoglycans in cancer, natural killer (NK) cell-mediated cytotoxicity and Th17 cell differentiation was reduced in these patients.

To identify up-regulated to increased expression of hallmark gene sets possibly involved in faster disease progression, comparisons of fast- versus slow-progressing patient groups at primary and metastatic levels were also carried out using pre-ranked Gene Set Enrichment Analysis (GSEA) (Figure 3D and Supplementary Figure S6, available at https://doi.org/10.1016/j.esmoop.2022.100741). GSEA suggests reduction of expression of apical junction complex in fast-progressing patients, along with myogenesis primarily in metastatic lesions. In this context, some immune related cytokines (i.e. interferon-α (IFN-α), IFN-γ, interleukin 6) also differed significantly in the fast-progressing patients compared with those with late BMs. Furthermore, several proliferation-related gene sets previously associated with metastasis progression (e.g. cell cycle related targets of E2F transcription factors, genes involved in G2/M checkpoint and genes regulated by Myc^45^, ^46^, ^47^, ^48^), as well as a gene set associated with unfolded protein response,^49^ were significantly up-regulated in BM samples from fast-progressing patients. The mTORC1 and Hedgehog signaling pathways also showed dysregulation; the former was significantly up-regulated whereas expression of the latter was reduced in BM samples from fast-progressing patients. Although epithelial-to-mesenchymal transition (EMT) pathways are known to be associated with metastasis development, we found a reduction of expression of these processes in both primary and metastatic tumors of fast-progressing patients (Supplementary Figure S7, available at https://doi.org/10.1016/j.esmoop.2022.100741).

Finally, we compared the patients who developed multiple BMs (primary *n* = 8 and metastases *n* = 8) with those presenting a single BM (primary *n* = 12 and metastases *n* = 11) (Supplementary Table S1, available at https://doi.org/10.1016/j.esmoop.2022.100741). These examinations resulted in only a few dysregulated KEGG pathways (Figure 3E and F, Supplementary Table S4, available at https://doi.org/10.1016/j.esmoop.2022.100741), of which up-regulation of the ribosome pathway was prevalent in both primary and metastatic lesions of patients with multiple BMs. In addition, in primary tumors of patients with multiple BMs, the lysosome pathway was also slightly up-regulated, whereas expression of cell cycle and the complement and coagulation cascades was significantly reduced. Furthermore, metastatic tumors of patients with multiple BMs revealed reduction expression of of the ECM receptor interaction and focal adhesion pathways.

### Verification of proteins associated with fast BM development

Based on the results from the enrichment analyses and a thorough literature search, four proteins (EPCAM, MUC4, HTRA2 and RAB25) were selected for further verification using a parallel reaction monitoring assay. In our discovery data, several mitochondrial ribosomal proteins (MRPs) showed increased expression in the primary tumors of patients with fast-progressing and multiple BMs. Therefore, we also included 11 MRPs (MRPL19, MRPL23, MRPL1, MRPS23, MRPS10, MRPS6, MRPL49, MRPS16, MRPL18, MRPL43 and MRPL47) in the assay (Supplementary Table S6, available at https://doi.org/10.1016/j.esmoop.2022.100741).

**Figure 4:**
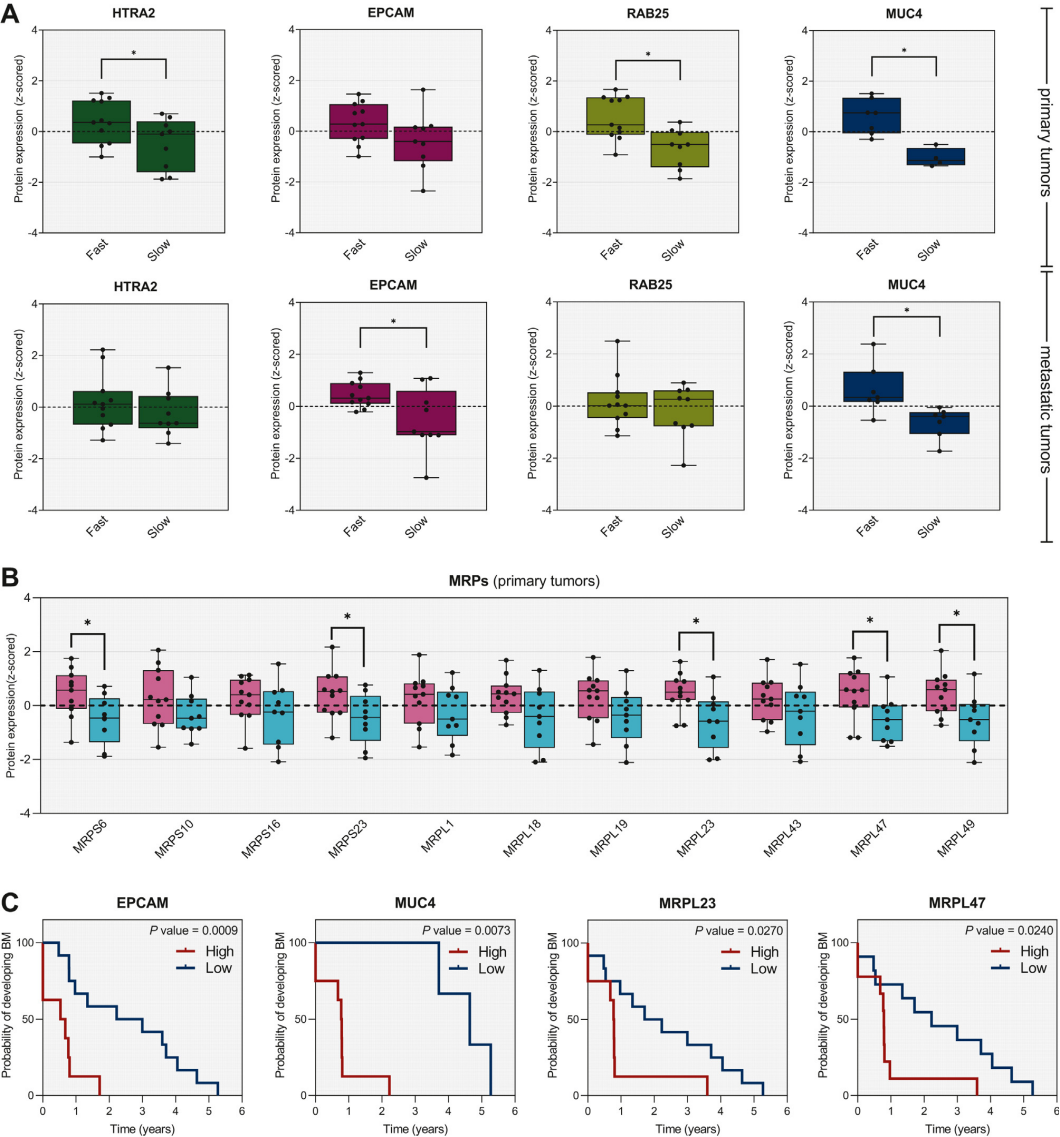
**Verification of potential biomarkers associated with fast progression to brain metastasis.** (A) Boxplots showing the z-scored protein expression of the four selected markers (HTRA2, EPCAM, RAB25, MUC4) between fast- and slow-progressing groups in primary (top) and metastatic tumors (bottom). (B) Boxplots showing the z-scored protein expression of the 11 MRPs (S6, S10, S16, S23, L1, L18, L19, L23, L43, L47, L49) between fast- (pink) and slow-progressing (turquoise) groups in primary tumors. (C) Kaplan–Meier plots of the four most promising biomarkers of fast brain metastatic progression: EPCAM, MUC4, MRPL23 and MRPL47 (*P* values of 0.0009, 0.0073, 0.0270 and 0.0240, respectively). High expression of these markers in primary tumors was associated with the development of BM within a shorter period of time. BM, brain metastasis; MRPs, mitochondrial ribosomal proteins.

In total, 10 out of the 15 markers showed significant differences in protein abundances between the fast- and slow-progressing groups in primary (fast *n* = 11 and slow *n* = 9) and/or metastatic brain (fast *n* = 11 and slow *n* = 8) tumors (Figure 4A and B). Among the selected candidates, MUC4 was significantly up-regulated in both primary and metastatic samples of fast-progressing patients, whereas HTRA2 and RAB25 were significantly elevated only in primary samples and EPCAM only in metastatic tumors of the fast-progressing group. The levels of all MRPs were higher in the primary tumors of fast-progressing patients (Figure 4B), among which MRPS6, MRPS23, MRPL23, MRPL47 and MRPL49 were significant.

In addition, Kaplan–Meier analysis was applied to predict the development of BM using the best cut-off point for each protein from the PRM results (Figure 4C). Increased expression of EPCAM (*P* value 0.0009), MUC4 (*P* value 0.0073), MRPL23 (*P* value 0.027) and MRPL47 (*P* value 0.024) showed a significant association with fast progression to BM, verifying the findings from the discovery data.

## Discussion

In this study, we aimed to identify proteomic signatures associated with primary and brain metastatic LADCs. Our results suggest that primary LADCs are characterized by a predominance of immune system-, cell-cell interaction- and migration-related pathways, whereas in corresponding BMs, metabolic, translation and vesicle formation pathways are enriched. Several proteins up-regulated in primary or metastatic tumors have known pro-metastatic features, such as COMP, CAV1, HMGB1 and YBX1.^41^ It has been described that COMP promotes EMT in colorectal cancer^50^ and contributes to disease severity in breast cancer.^51^ Overexpression of CAV1 has been correlated with advanced disease stage and shorter survival in LADC patients,^52^^,^^53^ whereas HMGB1 has been shown to be involved in proliferation and metastasis of LADC cells.^54^

Many of the proteins which positively correlated with tumor content both in primary and metastatic lesions have been previously described in the context of LADC.^36^ Of these, the YBX1 protein, which has been associated with poor prognosis and early metastasis in renal cell and hepatocellular carcinoma^41^ is noteworthy, together with the ribosomal proteins previously related to tumor development and other associated processes.^37^, ^38^, ^39^, ^40^ Additionally, histological examination showed that BMs exhibit a higher intratumoral vascularization, which is known to be a hallmark of the BM cascade.^55^ Indeed, preclinical data have already demonstrated that neoangiogenesis plays an essential role in BM formation in NSCLC.^56^ In other entities such as breast cancer, high vascularization has also been linked to increased metastatic potential and tumor progression.^57^

Importantly, to our knowledge, this is the first study investigating the proteomic landscape of both primary and metastatic LADC samples according to the onset of BMs. From a biological point of view, the significantly up-regulated pathways and pathways with reduced expression in the surgically resected samples of fast- versus slow-progressing patients might provide insights into the pathomechanism and even therapeutic possibilities of BMs. Notably, we found that expression of pathways related to cell-cell interactions was significantly reduced in fast-progressing patients. Of note, according to previous studies, dysregulation of focal adhesion and proteoglycans can impact metastatic events via regulation of intra- and extravasation, and the decrease of attachment abilities.^58^, ^59^, ^60^ Our results, therefore, suggest that malfunction of cell-cell adhesion and interaction contribute to metastasis. Pathways related to the immune system were also diminished in patients who developed BM within 1 year of LADC diagnosis. NK cells are critical for the control of metastatic dissemination since they contribute to the eradication of tumor cells and, moreover, participate in immunoediting of metastatic tumor cells.^61^^,^^62^ Accordingly, improved NK cell cytotoxicity, as observed in slow-progressing patients, has been associated with good prognosis in different cohorts of cancer patients.^61^ Additionally, the high number of Th17 cells, which might be as well specific for patients with late BMs, is also associated with improved survival outcomes in cancer patients of diverging entities.^63^ Our data provided hints that the immune system may also play a role in metastatic progression of LADC.

In our study, the fast-progressing tumors exhibited significant up-regulation of pathways associated with ribosomal activity along with metabolic reprogramming. Interestingly, the ribosome pathway was found to be enriched among proteins that correlated with tumor content in both tissue types, as well as in patients who developed multiple BMs. Ribosome biogenesis is a marker of tumor cell proliferation and is negatively associated with patient survival in p53-negative cancers.^64^ Moreover, it has been demonstrated that ribosome biogenesis is a common attribute of EMT.^65^ Genes coding for ribosomal proteins have already been linked to increased metastatic burden and aberrant expressions of ribosomal constituents which furthermore lead to altered translational efficiency.^66^ These conditions were less likely to occur in slowly progressing patients and in those with single BMs. Therefore, our results strongly suggest the contribution of ribosomal activity in tumorigenesis and metastasis formation in LADC.

Pathways related to metabolism were also overrepresented in fast-progressing patients, indicating their prominent role in BM development. Beyond cellular proliferation, aberrant cancer cell metabolism is closely related to cell fate and phenotype, followed by epigenetic changes and amended interactions of tumor cells with their surrounding environment.^67^ Certainly, cancer cells often profit from the modification of several core metabolic pathways such as glucose or lipid metabolism.^67^ Metabolic reprogramming in general is known to facilitate EMT, moreover enabling tumor cells to gain plasticity which is required for metastatic dissemination.^67^, ^68^, ^69^ Malignant cells that have gained increased invasive and migratory capacity frequently undergo metabolic reprogramming, which enables the cells to erode the ECM and extravasate into blood vessels. Required plasticity to grow in a different tissue also relies on metabolic reprogramming.^68^^,^^69^ Hence, pathways affecting the cellular metabolism and protein synthesis are crucial for early development of BMs.

GSEA results also emphasized the loss of epithelial organization as a hallmark of metastatic progression^70^ and suggested the involvement of reduction of expression of myogenesis. Muscle wasting has been previously linked to cancer mortality,^71^ as tumor-derived cytokines can play a role in myogenesis impairment and immune microenvironment alterations.^72^ IFN-γ is a key activator of cellular immunity and antitumor immune response^73^ and expression was found to be reduced in fast progression patients. In contrast, IFN-α, which has recently been associated with the most aggressive type of breast cancer contributing to migration processes,^74^ was up-regulated in fast-progressing patients. The reduction of expression of the EMT pathways is contradictory, however, loss of EGFR has been demonstrated after EMT induction and was correlated with metastatic process.^75^ In addition, overexpression of EGFR may be lost after LADC tumor cells have migrated to the brain.^76^ Regarding EMT markers, loss of FTSL1 has previously been associated with the metastatic potential of lung cancer cells,^77^ and poor prognosis in LADC patients, especially in smokers.^78^ Silencing of FLNa expression in lung cancer cell lines can promote proliferation, migration and invasiveness.^79^ And finally, the loss of pro-apoptotic factor Fas increases profibrotic functions in the lungs of idiopathic pulmonary fibrosis patients,^80^ which can predispose them to developing NSCLC. Moreover, up-regulation of proliferation markers associated with metastasis progression such as Myc,^45^, ^46^, ^47^ as well as mTORC1 and Hedgehog signaling may also be involved in faster metastatic development. Myc is considered a candidate driver of BMs in LADC, as higher amplification frequencies were previously reported in BMs compared with primary LADC tumors.^48^ Overexpression of mTOR complexes is known to be related with metastatic events, whereas mTORC1 plays a key role in regulating cell growth, cell proliferation, survival and motility.^81^ Hedgehog signaling is usually associated with tumorigenesis and metastatic events,^82^ but reduction of expression can also lead to brain damage and neuronal apoptosis.^83^^,^^84^ Altogether, GSEA has pointed out interesting pathways and markers that may be playing a role in faster metastatic progression in LADC patients.

Comparison of patients with multiple BMs versus single BM revealed scarcely any significant pathways. This may be partly due to the small number of heterogeneous samples (only four per group).

Finally, we verified the differential expression of nine potential biomarkers, including five MRPs between tumor samples of LADC patient groups with fast- versus slow-progressing BMs using a targeted PRM assay. EPCAM has been previously described to be highly expressed in rapidly proliferating carcinomas and to be involved in important processes related to proliferation.^85^ MUC4 can promote tumor growth by suppressing apoptosis and may be a valuable prognostic marker and therapeutic target.^86^, ^87^, ^88^ Higher mRNA expression of HTRA2 is associated with higher clinicopathological stage and worse prognosis in gastric cancer.^89^ RAB25 bound to Rab coupling protein (RCP) is known to increase cancer invasion and metastasis and the inhibition of RAB25-RCP is a potential therapeutic target.^90^ Several MRPs and their encoding genes have previously been linked to cancer.^91^

Like all retrospective analyses, our study has limitations. Thus, not all information concerning the patients' clinicopathological variables could be retrieved from the medical records. The relatively small cohort size and the lack of comparisons between metastatic and non-metastatic LADCs also constitute potential study limitations. Lastly, the threshold value used to differentiate fast- versus slow-progressing patients although clinically justified, is still somewhat arbitrary and its relevance needs to be further assessed in larger cohorts.

### Conclusions

Our results shed light on the specific proteomic profiles of primary LADCs and their corresponding BMs. These profiles were translated into biologically relevant pathways, thus contributing to a better understanding of disease progression, from migration-related pathways in primary tumors to metabolic reprogramming in BMs. Throughout our analyses, we observed a loss of cell-cell interaction- and immune system-related pathways in fast-progressing patients and in those with multiple BMs. Accordingly, these processes might facilitate metastatic spread to the brain and might influence the timing of these CNS metastases in LADC patients. Additionally, fast-progressing patients presented significant up-regulation of pathways associated with ribosomal activity along with metabolic reprogramming. Of note, an increase in ribosomal activity proved to be critically associated with tumorigenesis as well. Verification of our results revealed that EPCAM and MUC4 are promising biomarkers for BM progression in LADC, together with MRPL23 and MRPL47. By analyzing this unique cohort of surgically resected LADCs and their corresponding BMs with proteomics, our results provide insights into the biological processes involved in the metastatic spread, and moreover, might contribute to the development of novel personalized follow-up strategies in the clinics.
