# Supplementary material for: Proteomic analysis of brain metastatic lung adenocarcinoma reveals intertumoral heterogeneity and specific alterations associated with the timing of brain metastases
Source: ESMO Open. 2022 Dec 16;8(1):100741. doi: 10.1016/j.esmoop.2022.100741 (PMC10024110; doi:10.1016/j.esmoop.2022.100741)
Supplement: Supplementary Material [file mmc1.docx]

**Proteomic analysis of brain metastatic lung adenocarcinoma reveals intertumoral heterogeneity and specific alterations associated with the timing of brain metastases**

Nicole Woldmar^1,2*^, Anna Schwendenwein^3*^, Magdalena Kuras^4*^, Beáta Szeitz^5^, Kristiina Boettiger^3^, Anna Tisza^6,7^, Viktória László^3,6^, Lilla Reiniger^7,8^, Attila G. Bagó^9^, Zoltán Szállási^8,10,11^, Judit Moldvay^6,8^, Attila Marcell Szász^5,6^, Johan Malm^4^, Péter Horvatovich^12^, Luciana Pizzatti^2^, Gilberto B. Domont^13^, Ferenc Rényi-Vámos^6,14^, Konrad Hoetzenecker^3^, Mir Alireza Hoda^3^, György Marko-Varga^1^, Karin Schelch^3^, Zsolt Megyesfalvi^3,6,14^, Melinda Rezeli^1#^, Balázs Döme^3,4,6,14#^

^1^Department of Biomedical Engineering, Lund University, Lund, Sweden;

^2^Laboratory of Molecular Biology and Proteomics of Blood/LADETEC, Institute of Chemistry, Federal University of Rio de Janeiro, Rio de Janeiro, Brazil;

^3^Department of Thoracic Surgery, Medical University Vienna, Vienna, Austria;

^4^Section for Clinical Chemistry, Department of Translational Medicine, Lund University, Skåne University Hospital Malmö, Malmö, Sweden;

^5^Department of Bioinformatics, Semmelweis University, Budapest, Hungary;

^6^National Korányi Institute of Pulmonology, Budapest, Hungary;

^7^Department of Pathology and Experimental Cancer Research Semmelweis University, Budapest, Hungary;

^8^Department of Pathology, Forensic and Insurance Medicine, MTA-SE NAP, Brain Metastasis Research Group, Hungarian Academy of Sciences;

^9^Department of Neurooncology, National Institute of Clinical Neurosciences, Budapest, Hungary, Budapest, Hungary;

^10^Computational Health Informatics Program, Boston Children's Hospital, Harvard Medical School, Boston, Massachusetts, USA;

^11^Danish Cancer Society Research Center, Copenhagen, Denmark;

^12^Department of Analytical Biochemistry, Groningen Research Institute of Pharmacy, University of Groningen, The Netherlands;

^13^Department of Biochemistry, Institute of Chemistry, Federal University of Rio de Janeiro, Rio de Janeiro, Brazil;

^14^Department of Thoracic Surgery, National Institute of Oncology-Semmelweis University, Budapest, Hungary

*NW, AS and MK share the first authorship

^#^BD and MR contributed equally as senior authors to this study

**Supplementary Material**

**Table of Contents:**

**Supplementary Materials and Methods** S3

**Supplementary Figure 1.** Data preprocessing workflow for statistical analyses S9

**Supplementary Figure 2.** Characterization of the LADC cohort S10

**Supplementary Figure 3.** Characterization of the 6,821 quantified proteins S11

**Supplementary Figure 4.** Unsupervised clustering S12

**Supplementary Figure 5.** The number of quantified proteins in each sample group S13

**Supplementary Figure 6.** Gene Set Enrichment Analysis (GSEA) S14

**Supplementary Figure 7.** Volcano plot of the proteins belonging to the EMT pathway S15

**Supplementary Table 1.** Detailed clinical and histological information. Proteomic data with statistical test results.

**Supplementary Table 2.** Results from correlation analyses related to tumor content and necrosis score. Pathways associated with proteins correlating with tumor content in both primary and metastatic tissues.

**Supplementary Table 3.** Comparison of our data with known LADC- or metastasis-related proteins from the literature.

**Supplementary Table 4.** Results from 1D and 2D annotation enrichment analyses. Pathways associated with differentially expressed proteins between fast- and slow- progressing patients.

**Supplementary Table 5.** Tissue specificity analysis on both primary and metastatic tissues using databases from lung and brain tissues.

**Supplementary Table 6.** PRM assay details and results.

**Supplementary Materials and Methods**

***Sample preparation***

Primary LADC FFPE blocks were macro-dissected to deplete samples of adjacent tissue. Since brain metastasectomy aims to reduce the risk of neurocognitive deficit and is, therefore, usually very precise, no macrodissection was performed on BM samples. Therefore, even without macrodissection, BM samples mostly contained tumor and necrotic areas rather than adjacent tissue. FFPE blocks were sectioned for proteomic (2-3 sections of 30 µm thickness, cut directly into 1.5 mL tubes) and histopathological analyses (one section, taken consecutively). Histological sections were stained with H&E, scanned and then analyzed by a certified pathologist. Tumor area (%), adjacent tissue area (%), necrotic area (%), mucin production score (0-3), stromal density score inside the tumor (0-3), necrosis score inside the tumor (0-3), vascularization score (0-3) and lymphatic score (0-6) (combination of lymphocyte distribution and density) were calculated using the QuPath software ^1^ (Table 1). The scores were combined into low- vs. high-value groups for further statistical analysis (0-1/0-1 = low-values, 2-3/3-6 = high-values).

***Sample processing for proteomic analysis***

Processing of the FFPE samples for proteomics followed the main steps of deparaffinization, protein extraction and digestion (S-Trap), as previously described by our group ^2^. In brief, deparaffinization was performed by incubating the samples with 1:50 diluted EnVision™ FLEX Target Retrieval Solution High pH (Agilent Dako) at 97 ˚C for 10 min, followed by a brief centrifugation for the removal of the solution and the paraffin layer. Protein extraction buffer of 100 mM TEAB containing 25 mM DTT and 10 w/v% SDS (pH 8) was then added to the samples, and to induce antigen retrieval they were incubated at 99 ˚C for 1 h. Protein extraction was performed in a Bioruptor® Plus UCD-300 (Diagenode) by sonication for 40 cycles at 4 ˚C, followed by a high-speed centrifugation. Protein determination was performed according to the manufacturer’s instructions using a Pierce 660 nm Protein Assay (Thermo Scientific) supplemented with ionic detergent compatibility reagent (Thermo Scientific).

Digestion was carried out on the S-Trap™ 96-well Plate (ProtiFi) with 50 µg of proteins per sample, as previously described ^2^. Alkylation was performed with 50 mM iodoacetamide and stopped by the addition of 1.2 % phosphoric acid. Then, S-Trap binding buffer (90 % methanol, 100 mM TEAB) was added to the acidified lysates and the samples were loaded onto the S-Trap filters. The proteins were washed four times with S-Trap binding buffer and short centrifugations. Digestion buffer (50 mM TEAB) containing endoproteinase LysC at 1:50 w/w ratio (enzyme/protein) was added onto the filter and incubated for 2 h at 37 ˚C. Next, digestion buffer containing trypsin at 1:50 w/w ratio (enzyme/protein) was added on top and incubated overnight at 37 ˚C. On the following day, the peptides were eluted with the addition of digestion buffer, then 0.2 % aqueous formic acid, and finally, 50 % acetonitrile (ACN) containing 0.2 % formic acid. The eluted peptides were acidified with formic acid and dried down in a centrifugal evaporator.

***nLC-MS/MS analysis and database search***

The nLC-MS/MS analysis was performed on an Ultimate 3000 RSLC nano pump (Thermo Scientific) coupled to a Q Exactive HF-X (Thermo Scientific) mass spectrometer equipped with an EASY-Spray ion source. 1 µg of the peptides were loaded onto an Acclaim PepMap 100 C18 column (75 µm × 2 cm, 3 µm, 100 Å, nanoViper) and then separated on an Easy-spray PepMap RSLC C18 column (75 µm × 25 cm, 2 µm, 100 Å) (Thermo Scientific) using a flow rate of 300 nL/min and a column temperature of 45 °C. A 145 min non-linear gradient was applied using the solvents A (0.1 % formic acid) and B (0.1 % formic acid in 80 % ACN). The gradient increased from 2 to 25% of solvent B in 115 min, then to 32% of B in the next 10 min, further increasing to 45% in 7 min. At last, the gradient increased to 90% in 8 min, staying for another 5 min. The samples were analyzed using a top 20 DDA (data dependent acquisition) method with the following settings: full MS1 scans at m/z 375-1500, resolution of 120,000 (at 200 m/z), target AGC value of 3·10^6^ and maximum injection time of 100 ms; MS2 scans with a resolution of 15,000 (at 200 m/z), target AGC value of 1·10^5^, maximum injection time of 50 ms, fragmentation with NCE of 28, 1.2 m/z of isolation window, ion selection threshold of 8·10^3^ and dynamic exclusion of 40 s. All samples were run in duplicates and as a system performance check, 200 fmol of HeLa protein digest standard (Pierce) was run regularly.

Protein identification and quantitation were performed in Proteome Discoverer vs2.4 (Thermo Scientific) using Spectral Library (Proteome tools human spectrum library) and Sequence Database (SEQUEST HT) searches against the UniProtKB human database (downloaded 2019-01-15). The search implemented carbamidomethylation of the cysteine residues as a static modification; oxidation of methionine and acetylation on the protein N-terminus as dynamic modifications; 10 ppm precursor ion tolerance and 0.02 Da fragment ion tolerance. Two missed cleavages were acceptable for tryptic peptides and the minimum peptide length was set to six amino acids. The filter High Confidence (1 % FDR) was applied on both peptide and protein levels.

***Parallel reaction monitoring (PRM)***

Parallel reaction monitoring (PRM) was performed to verify a set of differentially expressed proteins. One to five unique peptides for each protein of interest were selected, and in the final assay, 39 peptides corresponding to 16 proteins (including GAPDH) were monitored (Supplementary Table 6). 1 µg of the peptides were loaded onto an Acclaim PepMap 100 C18 column (75 µm × 2 cm, 3 µm, 100 Å, nanoViper) and then separated on an Easy-spray PepMap RSLC C18 column (75 µm × 25 cm, 2 µm, 100 Å) (Thermo Scientific) using a flow rate of 300 nL/min and a column temperature of 45 °C. A 75 min gradient was applied for separation, using the solvents A (0.1 % formic acid) and B (0.1 % formic acid in 80 % ACN). The gradient increased from 2 to 25% of solvent B in 50 min, then to 32% of B in the next 6 min, further increasing to 45% in 4 min. At last, the gradient increased to 95% in 2 min, staying for another 10 min. PRM was used with an inclusion list of the selected precursors with 6 min retention time windows. MS2 scans were acquired using 30,000 (@ 200 m/z) resolution, the AGC target value was set to 2e5 and 50 ms maximum injection time was used. Fragmentation was performed with a normalized collision energy of 28. Samples were randomly analyzed, and each run was followed by a blank injection to avoid any residues from the previous sample.

All raw data generated were imported to Skyline v21.2 software (MacCoss Lab Software, Seattle, WA) for further data analysis. Peak integration was done automatically by the software, using Savitzky-Golay smoothing, and was manually inspected to confirm the correct peak detection. Peak identities were confirmed by inspecting dot product scores provided by Skyline between library spectra and measured transitions (dotp).

***Biostatistical analysis***

To compare the clinical and histopathological parameters of the different progression groups, we performed contingency table analyses and t-tests (p-value < 0.05).

For the evaluation of the proteomic data, following the log_2_-transformation of the raw protein intensities, the values were centered around the global median. After normalization, the MS replicates showed a high correlation and were therefore averaged. To correlate histological information with protein abundance, no prior filters were applied and the analyses were conducted on lung and brain tumors separately. Spearman correlation analysis (p-value < 0.05) was performed on proteins with at least three valid values (perfect fits, i.e. coefficients very close to 1 and -1 were excluded) to search for proteins that correlate well with tumor content. 1-way ANOVA with Tukey HSD posthoc tests (p-value < 0.05) were performed to delineate proteins that show differences in expression based on necrosis score. Only proteins that were present in at least 70% of the samples in at least two necrosis score groups were included in group comparisons. P-values were adjusted via the Benjamini-Hochberg method. Proteins with at least 70% valid values in either lung or brain tumors were kept and one outlier sample was removed for group comparisons based on tumor origin (primary vs. metastatic), timing (fast- vs. slow-progressing subgroups) and number of metastases (multiple vs. single BMs). These comparisons were performed using t-tests (FDR < 0.05 or p-value < 0.05) (Supplementary Figure 1). Clustering and PCA analyses were performed after removing all proteins with missing values, in the latter, the outlier sample was excluded.

Pathway analyses were carried out using 1D and 2D annotation enrichment analysis, which takes into account all identified proteins and their fold changes. Pre-ranked Gene Set Enrichment Analysis (GSEA) was performed using the clusterProfiler R package version 3.18.1. The hallmark gene set was considered, and proteins were ranked based on their absolute log_2_ fold-changes between the fast- and slow-progressing subgroups. Gene ontology enrichment analysis, subcellular location of proteins and other functional annotation were performed using PANTHER ^3,4^, DAVID 6.8 ^5,6^, STRING ^7^, KEGG (Kyoto Encyclopedia of Genes and Genomes) ^8^, the Human Protein Atlas ^9^ (downloaded 2021-05-03, <https://www.proteinatlas.org/humanproteome/tissue>) and MSigDB (Molecular Signatures Database Hallmark Gene Set Collection) ^10^ tools. Our data was further compared with datasets from other studies, such as Gillette et al. ^11^, Mei et al. ^12^, and the human cancer metastasis database (HCMDB) ^13^ (Supplementary Table 3).

The raw peptide intensities from the PRM data were log2-transformed, then converted to protein intensities by taking the mean of the measured peptide intensities, and finally normalized to the GAPDH intensity. After normalization, fast- and slow-progressing groups were compared using t-test (p-value < 0.05). For each protein, we determined the threshold value for classification into high and low expression groups (resulting in the highest sensitivity and specificity) using receiver operating characteristic (ROC) curve, then a Kaplan-Meier plot was created and the differences between groups were compared using the log-rank test. Statistical analyses were performed in Perseus ^14,15^, GraphPad Prism v.9 and R v. 4.0 ^16^.

**References**

1. Bankhead P, Loughrey MB, Fernández JA, et al. QuPath: Open source software for digital pathology image analysis. *Sci Rep*. 2017;7(1):16878. doi:10.1038/s41598-017-17204-5

2. Kuras M, Woldmar N, Kim Y, et al. Proteomic Workflows for High-Quality Quantitative Proteome and Post-Translational Modification Analysis of Clinically Relevant Samples from Formalin-Fixed Paraffin-Embedded Archives. *J Proteome Res*. 2021;20(1):1027-1039. doi:10.1021/acs.jproteome.0c00850

3. Thomas PD, Campbell MJ, Kejariwal A, et al. PANTHER: A Library of Protein Families and Subfamilies Indexed by Function. *Genome Res*. 2003;13(9):2129-2141. doi:10.1101/gr.772403

4. Mi H, Dong Q, Muruganujan A, Gaudet P, Lewis S, Thomas PD. PANTHER version 7: improved phylogenetic trees, orthologs and collaboration with the Gene Ontology Consortium. *Nucleic Acids Res*. 2010;38(suppl_1):D204-D210. doi:10.1093/nar/gkp1019

5. Huang DW, Sherman BT, Lempicki RA. Bioinformatics enrichment tools: paths toward the comprehensive functional analysis of large gene lists. *Nucleic Acids Res*. 2009;37(1):1-13. doi:10.1093/nar/gkn923

6. Huang DW, Sherman BT, Lempicki RA. Systematic and integrative analysis of large gene lists using DAVID bioinformatics resources. *Nat Protoc*. 2009;4(1):44-57. doi:10.1038/nprot.2008.211

7. Szklarczyk D, Gable AL, Nastou KC, et al. The STRING database in 2021: customizable protein–protein networks, and functional characterization of user-uploaded gene/measurement sets. *Nucleic Acids Res*. 2021;49(D1):D605-D612. doi:10.1093/nar/gkaa1074

8. Kanehisa M. KEGG: Kyoto Encyclopedia of Genes and Genomes. *Nucleic Acids Res*. 2000;28(1):27-30. doi:10.1093/nar/28.1.27

9. Uhlén M, Fagerberg L, Hallström BM, et al. Tissue-based map of the human proteome. *Science (80- )*. 2015;347(6220). doi:10.1126/science.1260419

10. Liberzon A, Birger C, Thorvaldsdóttir H, Ghandi M, Mesirov JP, Tamayo P. The Molecular Signatures Database Hallmark Gene Set Collection. *Cell Syst*. 2015;1(6):417-425. doi:10.1016/j.cels.2015.12.004

11. Gillette MA, Satpathy S, Cao S, et al. Proteogenomic Characterization Reveals Therapeutic Vulnerabilities in Lung Adenocarcinoma. *Cell*. 2020;182(1):200-225.e35. doi:10.1016/j.cell.2020.06.013

12. Mei Y, Yang J-P, Qian C-N. For robust big data analyses: a collection of 150 important pro-metastatic genes. *Chin J Cancer*. 2017;36(1):16. doi:10.1186/s40880-016-0178-z

13. Zheng G, Ma Y, Zou Y, Yin A, Li W, Dong D. HCMDB: the human cancer metastasis database. *Nucleic Acids Res*. 2018;46(D1):D950-D955. doi:10.1093/nar/gkx1008

14. Tyanova S, Temu T, Sinitcyn P, et al. The Perseus computational platform for comprehensive analysis of (prote)omics data. *Nat Methods*. 2016;13(9):731-740. doi:10.1038/nmeth.3901

15. Cox J, Mann M. 1D and 2D annotation enrichment: a statistical method integrating quantitative proteomics with complementary high-throughput data. *BMC Bioinformatics*. 2012;13 Suppl 1(Suppl 16):S12. doi:10.1186/1471-2105-13-S16-S12

16. Wu T, Hu E, Xu S, et al. clusterProfiler 4.0: A universal enrichment tool for interpreting omics data. *Innov*. 2021;2(3):100141. doi:10.1016/j.xinn.2021.100141

**Supplementary Figures**

**
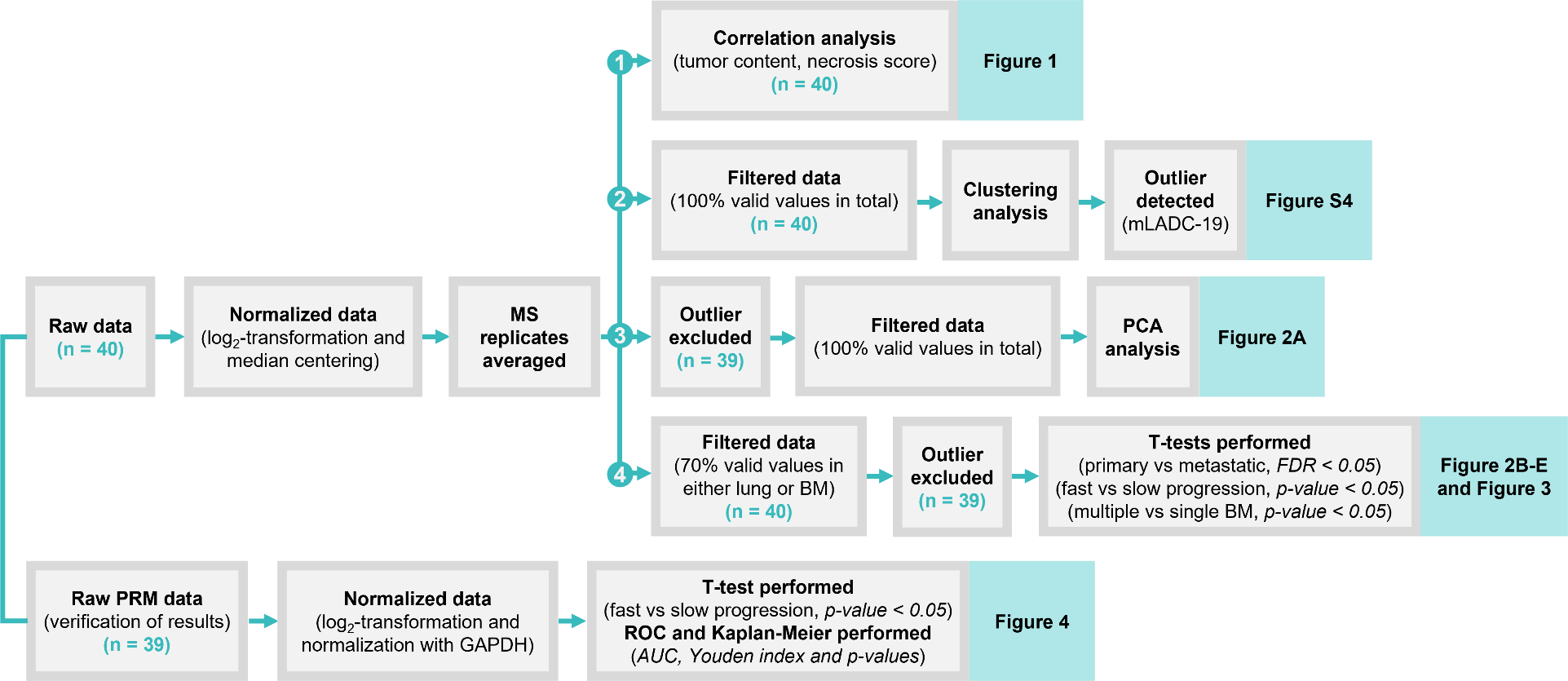
**

**Supplementary Figure 1.** **Data preprocessing workflow for statistical analyses.** Raw proteomic data was log_2_-transformed and median centered, followed by averaging of the MS replicates. Next, the following analyses were performed with the proteomic data. (1) Correlation with histological information conducted on primary and metastatic samples separately. Spearman correlation was performed for tumor content correlation, and 1-way ANOVA tests with Tukey HSD posthoc tests were used for necrosis score correlation. (2) Unsupervised clustering was performed on all 40 samples after 100% valid value filtering, detecting one outlier (mLADC-19). (3) PCA analysis was performed after outlier exclusion and 100% valid value filtering. (4) T-tests were performed on the data matrix containing proteins with at least 70% valid values in either primary or metastatic samples, excluding the outlier sample. In parallel, raw PRM data was obtained for the verification of selected candidate markers excluding the outlier sample. PRM data was log_2_-transformed and normalized to GAPDH signal, followed by t-test and ROC and Kaplan-Meier analyses.

**
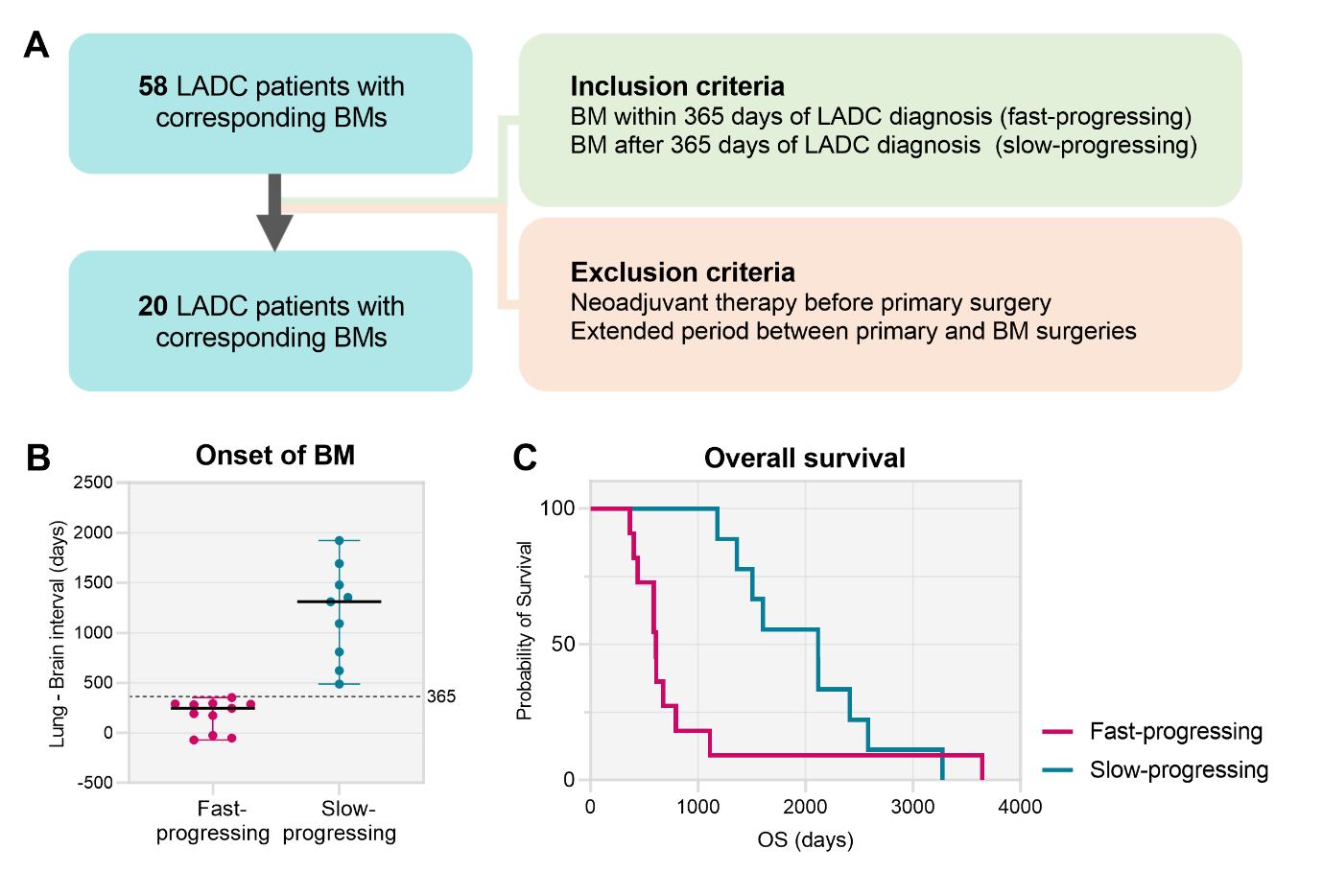
Supplementary Figure 2. Characterization of the LADC cohort.** (A) Flowchart for patient selection inclusion and exclusion criteria. (B) Onset of BM in all patients classified into fast- and slow- progressing groups (p-value < 0.0001; Mann Whitney test). (C) Overall survival of the fast- and slow-progressing groups. The colors pink and turquoise corresponds to fast- and slow-progressing groups, respectively.

**Supplementary Figure 3. Characterization of the 6,821 quantified proteins.** (A) Subcellular locations of the identified proteins based on the Human Protein Atlas database together with percentages. (B) Gene Ontology Biological Processes, (C) Gene Ontology Protein Classes and (D) Gene Ontology Molecular Functions of the identified proteins obtained from the PANTHER Classification System together with percentages. (E) Gene Ontology Biological Processes of differentially expressed proteins between primary lung (light orange) and metastatic brain samples (purple).

**
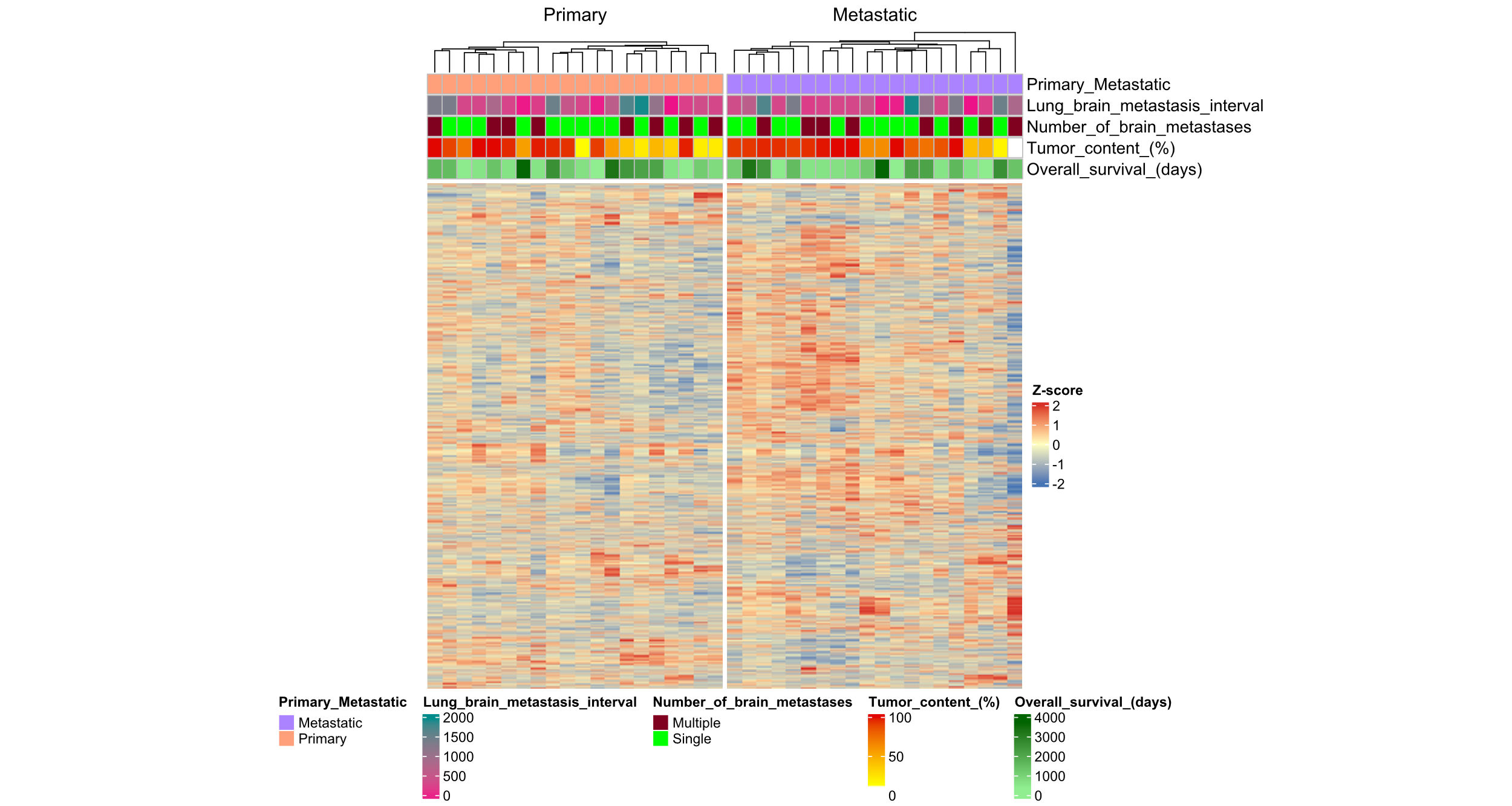
**

**Supplementary Figure 4. Unsupervised clustering.** Protein expression profile of the whole cohort, including the outlier brain metastasis sample on the right displaying low tumor content.

**
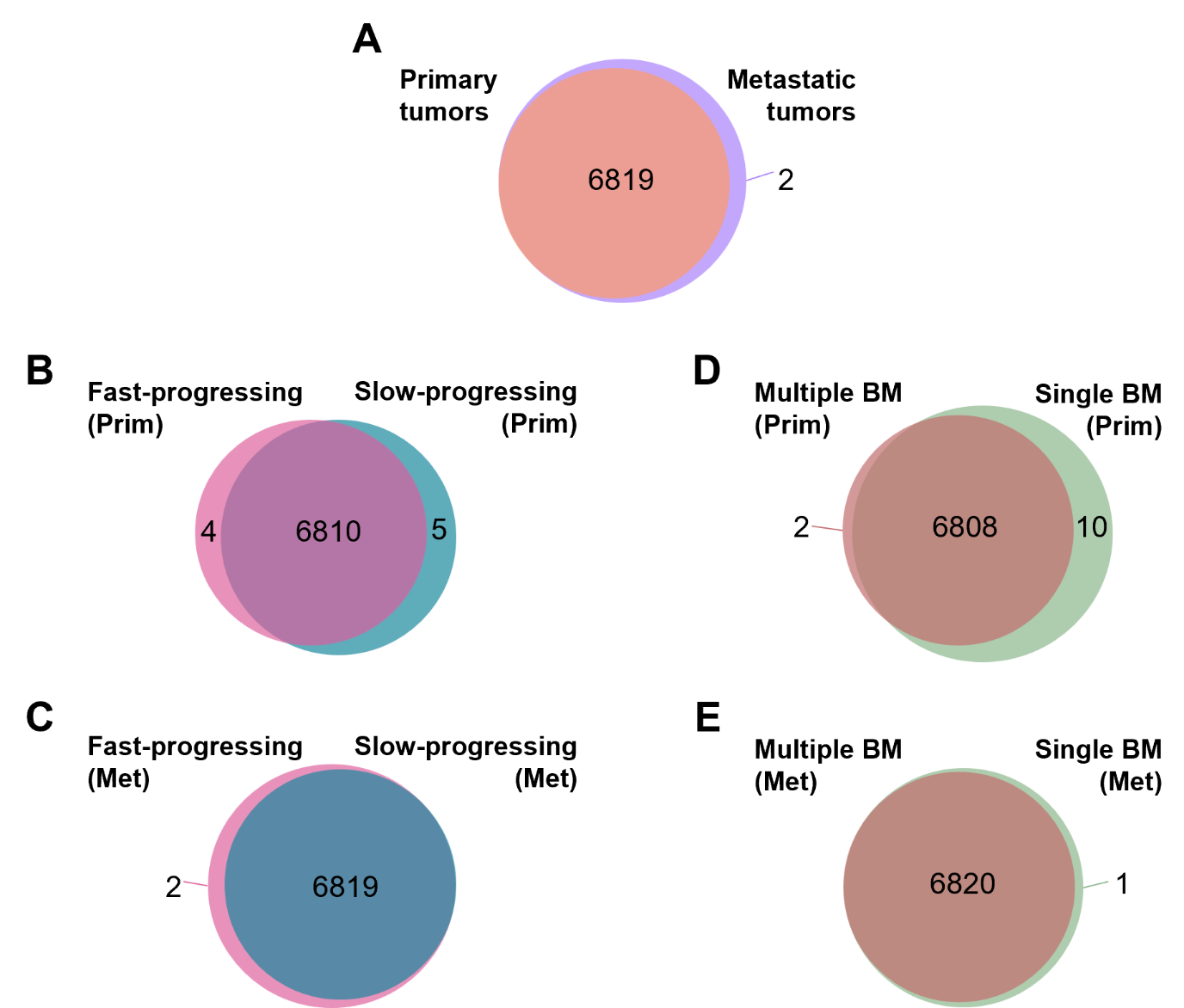
**

**Supplementary Figure 5. The number of quantified proteins in each sample group.** Venn diagrams showing the overlap of quantified proteins in the sample groups according to different comparisons: primary versus metastatic tumor samples (A), fast- versus slow-progressing patient tumors at the primary (B) and metastatic (C) levels, and samples of patients with multiple versus single BMs at the primary (D) and metastatic (E) levels. For the Venn diagrams we used a matrix containing proteins with at least 70% valid values in either the primary or metastatic samples, after excluding the outlier sample.

**
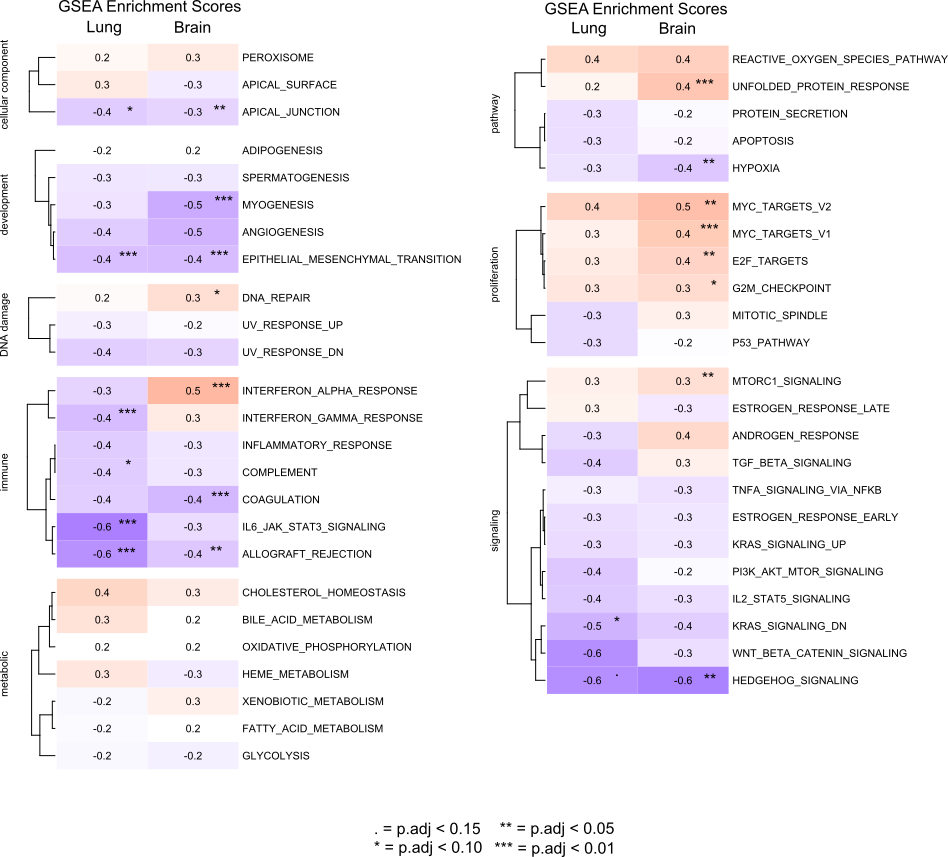
**

**Supplementary Figure 6. Gene Set Enrichment Analysis (GSEA).** Pathways upregulated (red) or downregulated (blue) in the primary lung or metastatic brain samples of fast-progressing patients.

**
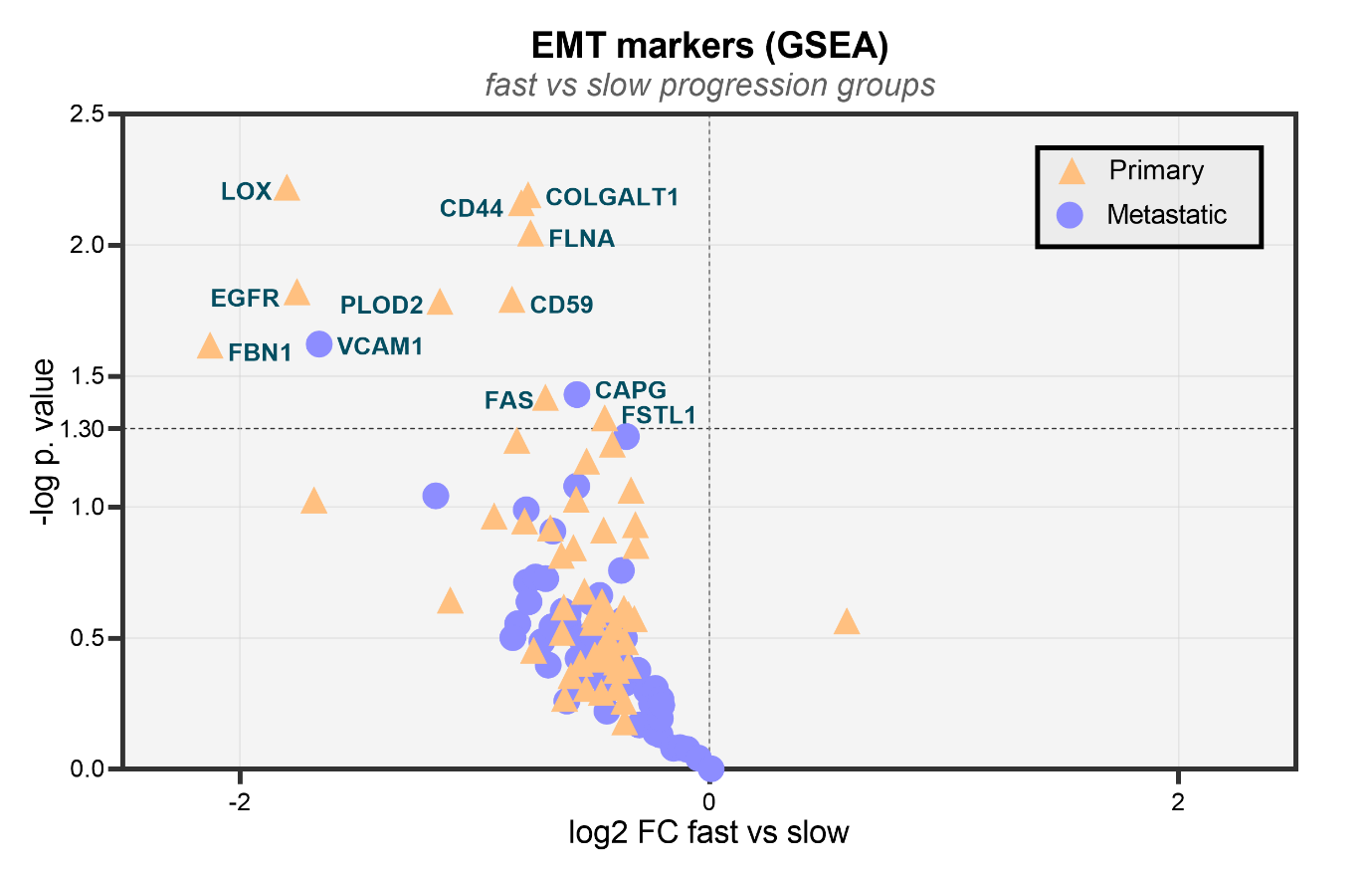
**

**Supplementary Figure 7.** **Expression of representative Epithelial-Mesenchymal Transition (EMT) markers from the GSEA analysis.** EGFR, PLOD2, FAS, CD59, FSTL1, FLNA, CD44, FBN1, COLGALT1 and LOX showed significantly different expression in fast- vs slow- progressing groups in primary tumors and VCAM1 and CAPG in metastatic tumors.
